# Twenty four microsatellite markers for *Aspidosperma polyneuron (Apocynaceae)*, an endangered tree species

**DOI:** 10.1186/1753-6561-5-S7-P7

**Published:** 2011-09-13

**Authors:** Ronai Ferreira-Ramos, Mariza Monteiro, Maria Imaculada Zucchi, José Baldin Pinheiro, Carlos Alberto Martinez, Moacyr Antonio Mestriner, Ana Lilia Alzate-Marin

**Affiliations:** 1 Programa de Pós-Graduação em Biologia Comparada, FFCLRP, Universidade de São Paulo (USP), Ribeirão Preto, SP, Brazil; 2 Departamento de Genética, ESALQ, USP, Piracicaba, SP, Brazil; 3 APTA Pólo Centro-Sul, Piracicaba, SP, Brazil; 4 Departamento de Biologia, FFCLRP, Universidade de São Paulo (USP), Ribeirão Preto, SP, Brazil; 5 Departamento de Genética, Faculdade de Medicina de Ribeirão Preto, USP, Ribeirão Preto, SP, Brazil

## Background

*Aspidosperma polyneuron* (Apocynaceae), commonly known as peroba-rosa, is a native and perennial species characteristic of semideciduous forest in submontane formation of Atlantic rain forest [1]. This species is recommended for ecosystems recovery and restoration of riparian forests in areas without flooding. But, the crescent logging, agriculture and urban expansion caused the reduction of native population of *A*. *polyneuron.* It is now included in the Red List of Threatened Species [http://www.iucnredlist.org/apps/redlist/details/32023/0]. Little is known about the population structure of *A. polyneuron* and the effects of the spatial isolation on the levels of genetic diversity and gene flow. In this study, we developed microsatellite loci for *A. polyneuron* aiming future studies of genetic structure, gene flow and mating system.

## Material and methods

Genomic DNA was extracted from leaf tissue [2] from one individual located in Cravinhos Municipality, São Paulo state, southeastern Brazil (21°17’41.8’’S; 47°42’08.5’’W). The Microsatellite-enriched library was constructed using the protocols described earlier [3] . The DNA was digested with *Rsa*I, bound to the adapters (Rsa21 and Rsa25) and amplified through polymerase chain reaction (PCR). The products were purified by Quiaquick PCR purification kit (Qiagen). Fragments containing microsatellite sequences were selected by hybridization with biotinylated oligo-probes (CT)_8_, (GT)_8_ and (CTT)_8_ and recovered by streptavidin-coated paramagnetic beads. After that, it was carried out a second PCR in order to increase the enriched fragments number and then an aliquot of the PCR product was bound to the pGEM-T vector (Promega) and transformed into *Escherichia coli* XL-1 Blue strains. The clones were sequenced using the Big Dye Terminator Kit and ABI 377 sequencer (Applied Biosystems) and the primers were designed using PRIMER3 [http://frodo.wi.mit.edu/primer3/].

Thirty *A. polyneuron* individuals were used for screening and the amplification were carried out in 10 μL reactions containing 2.5 ng of template DNA, 0.3 μM of each primer, 0.25 mM of each dNTP, 1x PCR buffer [75 mM Tris-HCl pH 9.0, 50 mM KCl and 20 mM (NH_4_)_2_SO_4_], 1.5 mM MgCl_2_ and 1U *Taq*DNA polymerase (Biotools); and performed using a MasterCycler^®^ (Eppendorf): 96°C for 4 min followed by 30 cycles (94°C for 40 s, 50-54°C for 1 min and 72°C for 1 min) and 72°C for 7 min. PCR products were denatured and separated on 8% denaturing polyacrylamide gels stained with silver nitrate. Allele sizes were estimated by comparison by 10 bp DNA ladder standard (Invitrogen) and the original DNA used for library development. FSTAT software [http://www2.unil.ch/popgen/softwares/fstat.htm] was used to calculate the genetic parameters per locus, alleles number, the genetic diversity (*H_e_*) and the linkage disequilibrium. The cumulative exclusion probabilities were calculated by CERVUS 3.0 software [4].

## Results and conclusions

A total of 384 clones were obtained and sequenced. Thirty-nine clones (10.2%) presented microsatellite repeats and 25 were suitable for primers design. The most common repeat motif was GA/CT as expected due the employing technique. Among the loci analyzed, one did not amplify, eight were monomorphic and 16 loci showed polymorphism in this sample. Twenty loci were dinucleotide (83.3%), one was tetra-, other one penta- and two were rare compounds loci: di/tri- (Apn8) and di/tetra- (Apn9). Mendelian inheritance analyses were carried out for each polymorphic locus using one mother tree and their open-pollinated family (26 progeny). All sibs displayed at least one of the maternal alleles, confirming Mendelian inheritance. From a total of 121 alleles presented in the sample, the genetic diversity was high (*H_e_* = 0.65) and the linkage disequilibrium, applying Bonferroni correction for multiple comparisons, was not significant. The total paternity exclusion probabilities over 16 loci were very high for one (*P*_*exclu*(*1*)_ = 0.9995) or both parents available (*P*_*exclu*(*2*)_ = 0.9999) indicating that the set is suitable for paternity analysis. Our study provides a new set of variable microsatellite loci for *A. polyneuron* that may be used to estimate genetic parameters such as genetic diversity, population structure, gene flow, and mating system. These markers might also prove transferable for genetic analyses in related taxa.

Financial Support: FAPESP, CNPq, CAPES-PROEX, FAEPA
